# 2022 update of the Austrian Society of Rheumatology and Rehabilitation nutrition and lifestyle recommendations for patients with gout and hyperuricemia

**DOI:** 10.1007/s00508-022-02054-7

**Published:** 2022-07-11

**Authors:** Judith Sautner, Gabriela Eichbauer-Sturm, Johann Gruber, Raimund Lunzer, Rudolf Johannes Puchner

**Affiliations:** 1 State hospital Korneuburg-Stockerau, 2. Med. Department of internal medicine, Lower Austrian Centre for rheumatology, Karl Landsteiner Institute for clinical rheumatology, Landstraße 18, 2000 Stockerau, Austria; 2 Private Practice of internal medicine, rheumatology and nephrology, Linz, Austria; 3 University hospital of internal medicine II, Med. University of Innsbruck, Innsbruck, Austria; 4 Department of internal medicine, Hospital of St. John of God Graz, Graz, Austria; 5 Private Practice of internal medicine, rheumatology and gastroenterology, Wels, Austria; 6 Medical University of Vienna, Vienna, Austria

**Keywords:** Gout, Recommendations, Nutrition, Lifestyle, Eduction

## Abstract

**Background:**

Gout is the most frequent inflammatory joint disease in the western world and has a proven genetic background. Additionally, lifestyle factors like increasing life span and wealth, sufficient to excess nutritional status and a growing prevalence of obesity in the population, as well as e.g. alcohol consumption contribute to the rising incidence of hyperuricemia and gout. Apart from an adequate medication, medical advice on nutrition and lifestyle is an essential part of the management of gout patients, being at high risk of internal comorbidities.

**Objective:**

In 2015, the ÖGR (Österreichische Gesellschaft für Rheumatologie und Rehabilitation) working group for osteoarthritis and crystal arthropathies already published nutrition and lifestyle recommendations for patients with gout and hyperuricemia. Since then, a multitude of literature has been published addressing this topic, what required an update.

**Methods:**

First, the authors performed a hierarchical literature search to screen the meanwhile published literature. Also considering references of the first publication, the relevant literature was selected, and the 2015 recommendations were either kept as published, reformulated or newly produced. Finally, the evidence level and the level of agreement with each recommendation were added.

**Results:**

Following this process, ten recommendations were generated instead of the initial nine. Like in the original publication, a colored icon presentation was provided to complement the written text.

**Conclusion:**

The Austrian nutrition and lifestyle recommendations for patients with gout and hyperuricemia were updated incorporating the most recent relevant literature, serving as education material for patients and updated information for physicians.

## Introduction

With a prevalence of 2–3%, gout is the most common inflammatory joint disease found among populations in Europe and North America, showing increasing incidence with age and wealth. This means gout is not just a general medical and rheumatological joint and pain therapy issue, but also an issue of increasing socioeconomic relevance [1]. Gout has the potential for chronicity and consecutive joint destruction and is also associated with cardiometabolic renal conditions, including hypertension, myocardial infarction, stroke, obesity, type 2 diabetes, hyperlipidemia, and chronic kidney disease [2, 3]. A number of recommendations are available from various rheumatological societies on both the diagnosis and treatment of gout [4–8]. Together with medication for sufficient attack management, attack prophylaxis and serum uric acid (SUA) reduction, advice on optimized nutrition and lifestyle are the cornerstones of patient care for those living with gout and hyperuricemia. Since the disease burden of gout correlates well with the sociodemographic index, nutrition and lifestyle become topics that cannot be ignored in an optimal and thorough gout management protocol [9].

Apart from prescribing medication, recommendations on diet and lifestyle modification have become integral to current guidelines for gout management. Despite ongoing discussions over the years, asymptomatic hyperuricemia is still not an indication for SUA-lowering therapy from a rheumatological point of view, because to date there is no evidence that the benefit of SUA lowering in this patient group outweighs the potential risks associated with certain pharmacological therapies. This underlines the potential and importance of including specific dietary and lifestyle recommendations to induce and support non-medication SUA reduction strategies in an ever-growing patient population with hyperuricemia (due to age, obesity, affluence, etc.). An additional and equally important aspect is the potential positive influence of dietary adjustments on internal comorbidities.

With optimal drug treatment and corresponding therapy adherence by patients, gout can in principle be successfully treated and even cured. In reality, however, sufficient treatment is lacking [10]. It is now widely accepted that treatment cannot succeed without a corresponding change in dietary behavior and the reduction of excess weight, which clearly indicates a non-pharmacological component of health management.

Recommendations, especially when they concern basic needs such as nutrition, only gain traction when adapted to local or cultural conditions. The regular updating of evidence-based recommendations is clearly indicated and becomes apparent in practice, especially in rheumatology.

## Material and methods

In 2014, the working group for osteoarthritis and crystal arthropathies within the Austrian Society for Rheumatology and Rehabilitation (ÖGR) produced and published dietary recommendations for patients with gout and hyperuricemia [11].

Due to an abundance of new literature since the 2014 publication, it became necessary to update these recommendations. The authors, representing five members of the working group, agreed to resume the hierarchical literature search and to work on the update. The literature search process was further supported by a medical journalist.

In the period from March to June 2021, 160 papers with publication dates up to 2021 were identified via a structured search via PubMed; 32 publications were added to this cohort since the last literature search in 2014. Of these, the topic of dietary approaches to stop hypertension (DASH) diet represented the largest proportion of newly published articles. 59 papers met the criteria for shortlisting after a more thorough review applying Oxford grading. Furthermore, recent papers generally addressing the impact of lifestyle changes, diet and weight with relevance in this context were added after completion of the literature search [60–62].

All studies were categorized by the authors according to their study design/evidence level (Oxford GRADE system) (Table 1). Based on this literature search and selection, the 2014 recommendations were assessed by the authors for their relevance in light of newly added literature, were graded according to the Oxford GRADE system and either kept as published, reformulated or newly produced [12]. Reviews or meta-analyses were used for several recommendations in cases of topic overlap. The authors concluded the paper with 10 recommendations compared to the previous 9 (Table 2). After group discussion, the authors felt it made more sense in terms of style to regroup and continue with general recommendations on weight regulation and dieting, followed by related negative and positive recommendations.

**Table 1:** Levels of evidence according to the Oxford Center for Evidence-based Medicine

Grade of recommendation	Level of evidence	Type of study
A	1a	Systematic review of (homogenous) randomized controlled trials
	1b	Individual randomized controlled trials (with narrow confidence intervals)
B	2a	Systematic review of (homogenous) cohort studies of “exposed” and “unexposed” subjects
	2b	Individual cohort study/low-quality randomized controlled trials
	3a	Systematic review of (homogenous) case-control studies
	3b	Individual case-control studies
C	4	Case series, low-quality cohort or case-control studies
D	5	Expert opinions based on nonsystematic reviews of results or mechanistic studies

**Table 2:** 10 recommendations on lifestyle and nutrition for patients with gout and hyperuricemia, recommendation 1–3: general, 4–7: nutritional dont’s, 8–10: nutritional do’s

1	Weight gain and obesity can lead to an increase in SUA levels and gout. In the case of adiposity, gradual weight loss (at least in men) can help lower SUA levels and thus protect against gout	
	*Evidence 2b (Grade B)*	*Level of agreement: 10*
2	Both gout and hyperuricemia are associated with cardiometabolic and renal comorbidities. Therefore, regular physical exercise/cardiovascular training (150(–300) min/week of moderate intensity) is recommended in addition to weight control and dietary measures	
	*Evidence 2a (Grade B)*	*Level of agreement: 10*
3	A healthy diet such as the dietary approaches to stop hypertension (DASH) diet, in combination with weight reduction if the patient is overweight, can positively influence gout incidence, elevated SUA levels and cardiometabolic risk	
	*Evidence 2b (Grade B)*	*Level of agreement: 9.8*
4	Red meat, offal and sausage products can increase SUA levels and thereby increase the risk of gout. For this reason, red meat and associated products should be eaten less frequently (2 ×/week) and only in small quantities. The consumption of purine-rich vegetables is explicitly recommended	
	*Evidence: 2b (Grade B)*	*Level of agreement: 10*
5	Seafood (especially crustaceans and mussels) can increase SUA levels and therefore the risk of gout and should therefore be consumed sparingly. Fish is recommended for consumption on a regular basis (1–2 ×/per week) as part of a generally healthy diet and also to help avoid cardiovascular disease	
	*Evidence: 3 (Grade B)*	*Level of agreement: 10*
6	Drinking alcohol increases the risk of gout in a dose-dependent manner. Beer and spirits in particular should be avoided, while red wine has the least potential for increasing the risk of gout	
	*Evidence: 2a (Grade B)*	*Level of agreement: 10*
7	Sugary soft drinks, fruit juices and high-fructose foods (fruit sugars) can increase SUA levels and should therefore be avoided. Fresh fruit and fructose-free “light drinks” do not increase the risk of gout	
	*Evidence: 3 (Grade B)*	*Level of agreement: 9.8*
8	Regular consumption of (low-fat) milk/dairy products can lower SUA levels and is recommended for all gout patients	
	*Evidence 1b (Grade A)*	*Level of agreement: 9.8*
9	Regular consumption of coffee can help to lower SUA levels—in combination with proper diet and medication and is therefore to be advocated	
	*Evidence: 2b (Grade B)*	*Level of agreement: 9.6*
10	Cherries (especially the Montmorency variety) can lower SUA levels by promoting urinary excretion. However, it is still unclear at what dose the different products (juice, concentrate, extract) yield the most desirable effect. It is possible that sour cherries in combination with allopurinol have a complementary effect	
	*Evidence 2b (Grade B)*	*Level of agreement: 9.0*

After formulation, the level of agreement from 1–10 (where 1 = no agreement and 10 = full agreement) was collected and averaged with each recommendation by all authors.

**Fig. 1:**
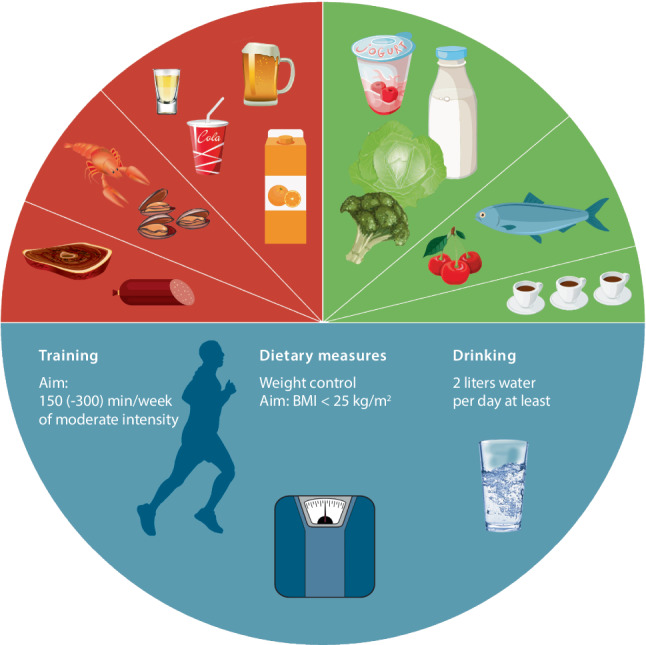
Nutrition- and lifestyle recommendations for patients with gout and hyperuricemia (courtesy of the ÖGR working group for osteoarthritis and crystal arthropathies; supported by Kwzida Pharma)

The graphical representation of the recommendations put forth in 2014, including icons for more effective message transmission and non-verbal communication with patients and their relatives, was adapted accordingly. Fig. 1.

## Results

1. Weight gain and obesity can lead to an increase in SUA levels and gout. In the case of adiposity, gradual weight loss (at least in men) can help lower SUA levels and thus protect against gout.
**Evidence 2b (grade B) | level of agreement: 10.**

### Comment

This recommendation was considered current and relevant and was adopted as it stands. The evidence has increased from 3 to 2b due to the availability of new topical literature. Observations from North American cohorts show a clear association between obesity and gout, both in men and women. The prevalence increases with degree of obesity. From large scale observational studies it can be concluded that in men with gout and obesity weight loss is a key factor in controlling gout [13, 14]. In a large-scale study of over 12,000 men, weight loss was shown to help normalize SUA levels in men with a high cardiovascular risk. Weight loss produced a more favorable SUA reduction than drug therapies, with additional health benefits [15]. A BMI of ≥ 25 kg/m^2^, alcohol consumption, non-adherence to a DASH diet and diuretic use were associated with hyperuricemia in a dose-dependent manner in this US study of 14,625 adults; however, the corresponding variance in serum urea explained by these risk factors was very small and, paradoxically, failed to demonstrate their high prevalence for assessing risk factors in practice [16].

2. Both gout and hyperuricemia are associated with cardiometabolic and renal comorbidities. Therefore, regular physical exercise/cardiovascular training (150 (–300) min/week of moderate intensity) is recommended in addition to weight control and dietary measures.
**Evidence 2a (grade B) | level of agreement: 10.**

### Comment

The wording of the present recommendation has been readapted from the 2014 publication and the level of evidence has increased from 3 to 2a.

Gout is closely associated with cardiovascular and metabolic diseases [2, 3]. It is also closely associated with insulin resistance and is considered part of the metabolic syndrome. But gout or hyperuricemia are not only associated with cardiometabolic and renal comorbidities; gout patients also show increased mortality. It is therefore not surprising that the determination of SUA levels as part of the work-up for arterial hypertension is recommended in the current guidelines of the European Society of Cardiology (ESC) [17]. Basically, aiming at a reduction of the cardiovascular risk in these patients, thereby promoting weight loss and SUA reduction, the amount and intensity of recommended physical exercise was indicated according to the aforementioned ESC guidelines. Leaving the type of exercise to the patient’s discretion, is a means to enhance individual diligence. Just like patients with cardiovascular diseases, patients with gout and hyperuricemia should engage in regular cardiovascular exercise [15, 16]. Apart from targeting SUA reduction, regular exercise is also to be strongly supported from a general internal medicine practitioner’s point of view.

3. A healthy diet such as the dietary approaches to stop hypertension (DASH) diet, in combination with weight reduction if the patient is overweight, can positively influence gout incidence, elevated SUA levels and cardiometabolic risk.
  **Evidence 2b (Grade B) | Level of agreement: 9.8.**

### Comment

This recommendation is new due to the abundance of more recently published literature on the DASH diet, with an evidence level of 2b.

The importance of an appropriate diet in gout and hyperuricemia is undisputed [18]. Differentiated drug therapies (including attack management, attack prophylaxis and SUA reduction) are included in all current recommendations for gout treatment; the importance of nutritional counselling as an important therapeutic component is mentioned in passing in the literature but is generally underrepresented.

Insulin resistance reduction is one of the significant effects of a weight control regimen even as weight reduction in general provides relief from a variety of obesity-related symptoms. A DASH diet also seems to have a SUA lowering effect in patients with gout and hyperuricemia [19]. In this context, one important study is devoted to patient education. It was able to show that comprehensive nutritional counselling leads to a significant improvement in patients’ knowledge as they participate in SUA-lowering therapy when dealing with gout; however, no difference could be found in SUA levels compared to the control group, which received only basic advice regarding adherence to the therapy and information on the benefits of weight loss [20]. In any case, nutritional counselling seems to have a positive influence on patients’ well-being.

Even diets that are not primarily focused on low purine content, such as the Mediterranean diet, calorie reduction, low-carb and low-fat, can likely also trigger a reduction in SUA and blood lipids (cholesterol and triglycerides) [21]. Reducing insulin concentrations via weight reduction and thereby a reduction in insulin resistance, thus positively influences cardiovascular risk factors [22]. From epidemiological observations, four significant factors contributing to the development of gout have emerged: Obesity, dietary behavior, alcohol consumption and use of diuretics. Healthy diets such as a Mediterranean diet combined with weight loss in the overweight and obese clearly lead to reducing these risk factors [23, 24]. A DASH diet also seems to have an identical effect [25]. The positive influence of a DASH diet on the reduction of SUA levels seems to be particularly effective in > 50-year-olds, in women in general and among those with limited physical activity [26]. With respect to the temporal dimension of the effect of a DASH diet, the SUA-lowering effect seems to occur after only 1 month and to last for at least 3 months. This effect is more pronounced the higher the initial SUA level [19]. The parameter that was initially decisive for the development of this form of diet, namely the blood pressure-lowering effect of a DASH diet, is also a positive factor for gout patients. In view of the literature, a DASH diet could be described as an attractive preventive diet for men at risk of gout [27]. In another study, the SUA-lowering effect of a DASH diet was confirmed, especially in the case of pre-existing hyperuricemia. Increased sodium intake also proved to lower SUA here, this phenomenon would require further studies to assess more thoroughly [28].

The question of whether age plays a role in dietary interventions was explored in the following study: a Mediterranean diet was found to be inversely associated with SUA levels in a cohort of older people (> 75 years) unafflicted by cardiovascular disease, however, reaching significance only in men (*p* = 0.02), confirming the cardioprotective effect of this diet [23]. The study also found that the diet was associated inversely with SUA levels, but significantly so only in men (*p* = 0.02).

4. Red meat, offal and sausage products can increase SUA levels and thereby increase the risk of gout. For this reason, red meat and associated products should be eaten less frequently (2 ×/week) and only in small quantities. The consumption of purine-rich vegetables is explicitly recommended.
  **Evidence: 2b (Grade B) | Level of agreement: 10.**

### Comment

This recommendation from 2014 was considered by the authors to be current and justified and was adopted almost unchanged. More recent literature has increased the evidence level from 3 to 2b.

According to the current literature, the connection between nutrition and gout risk management is essentially independent of gender, thus the recommendations apply equally.

Consumption of red meat (beef, lamb, pork) brings with it a higher risk of gout: multivariate relative risk (RR) = 1.41 (95% CI, 1.07–1.86; *p* = 0.02) [29–32]. An essential finding is the distinction between animal and vegetable purines and the benefits of all vegetable purines, the consumption of which should be encouraged in any case [33].

5. Seafood (especially crustaceans and mussels) can increase SUA levels and therefore the risk of gout and should therefore be consumed sparingly. Fish is recommended for consumption on a regular basis (1–2 ×/per week) as part of a generally healthy diet and also to help avoid cardiovascular disease.
  **Evidence: 3 (Grade B) | Level of agreement: 10.**

### Comment

This recommendation was classified as current and relevant as well and adopted unchanged. The evidence level remained the same as in 2014 at 3.

For seafood and crustaceans, the data are almost identical to those for red meat ([RR] = 1.51; 95% [CI] 1.17–1.95; *p* = 0.02). Overall, there was an increased risk of SUA elevation with consumption of seafood, except most fish, the exception being fatty fish, such as mackerel and sardines and all fish skin [30, 31]. To promote a healthy cardiovascular diet, this recommendation also differentiates in this respect with a clearly positive recommendation for fish consumption. An important aspect in these studies is dosing—in addition to infrequent consumption, smaller amounts should also be advised [32, 33].

6. Drinking alcohol increases the risk of gout in a dose-dependent manner. Beer and spirits in particular should be avoided, while red wine has the least potential for increasing the risk of gout.
  **Evidence: 2a (Grade B) | Level of agreement: 10.**

### Comment

This recommendation was classified as still current and relevant and adopted from the 2014 study using the same wording. More recent literature increased the level of evidence from 3 to 2a.

A large prospective observational study in the USA, with a follow-up of 26 years in 44,654 men with no history of gout, showed that the risk of gout rose with increasing alcohol intake (RR at ≥ 30.0 g/day 2.10) [34]. The evidence here is quite clear.

Among the different types of alcohol, the strongest association to risk of gout is that of beer, followed by spirits, according to more recent literature. In a widely accepted study by Choi et al. wine was not associated with an increased risk of gout [35].

Compared with teetotalers, the multivariate RR for men drinking 2–3 beers (1 beer = 335 ml)/week is 1.27 (95% CI 1.00–1.62), and the RR increases with increasing beer consumption (*p* < 0.0001). For beer consumption of ≥ 2 beers (corresponding to ≥ 670 ml)/day, the RR increases to 2.51 (95% CI 1.77–3.55) The multivariate RR for an increase of 1 beer/day is 1.49 (95% CI 1.32–1.70). A Japanese study examined different types of beer using the chromatography method, whereby locally produced beer (private breweries) and also non-alcoholic beer turned out to be particularly rich in purine; low-purine and low-malt beer carried the lowest risk of gout in this category [36]. In summary, alcohol, along with meat consumption, shows a linear relationship between frequency of intake, quantity and gout risk. Compared with teetotalers, the age-adjusted RR is 1.3 for alcohol consumption of 5–9.9 g/day and increases to 3.02 for consumption of 50 g/day (*p* < 0.0001) [16, 34, 35].

A recent systematic review dealt with smoking and alcohol consumption in patients with rheumatic and musculoskeletal diseases (RMD), amongst others with gout. While tobacco use does not seem to have an influence on gout attacks or SUA, alcohol consumption showed a significant association between the number and type of alcoholic beverages and the occurrence of flares [37]; however, to account for cultural differences in this context studies focusing on ethnicity and geographical residence are still lacking but needed.

7. Sugary soft drinks, fruit juices and high-fructose foods (fruit sugars) can increase SUA levels and should therefore be avoided. Fresh fruit and fructose-free “light drinks” do not increase the risk of gout.
  **Evidence: 3 (Grade B) | Level of agreement: 9.8.**

### Comment

The wording of this recommendation from 2014 was adapted and adjusted to reflect conclusions from the new literature. Fresh fruit was explicitly assessed as positive, also with regard to its role in a Mediterranean diet and the focus was expanded beyond foodstuffs to beverages in order to cover a broader range of convenience products containing fructose. The level of evidence has remained the same at 3.

Observational studies on > 89,000 subjects show that fructose significantly increases SUA levels and should therefore be avoided [38–42]. In view of the now almost ubiquitous addition of fructose apart from sweets, convenience foods in particular have a high risk potential in this respect [43, 44]. Fructose-rich fruit juices (especially orange juice) and sweet fruits (e.g. oranges or sweet apples) should be particularly avoided. In contrast, light and diet drinks without fructose are harmless in terms of gout risk. Other important aspects, such as the risk of developing diabetes mellitus, were deliberately not discussed here.

8. Regular consumption of (low-fat) milk/dairy products can lower SUA levels and is recommended for all gout patients.
  **Evidence 1b (Grade A) | Level of agreement: 9.8.**

### Comment

This recommendation from 2014 was classified as current and relevant and adopted with the same wording. The evidence remains the same as in 2014 with 1b.

As in 2014, this topic has the highest level of evidence (1b) of all the studies selected for these recommendations. [31, 32].

Several randomized trials have demonstrated the positive effect of milk on lowering SUA levels or gout per se [29, 30, 45–47]. The consumption of 250 ml of milk/day led to a 50% reduction in the risk of gout in men. Regular consumption of low-fat milk and yoghurts led to a 10% reduction in SUA levels. The reason for this positive impact is the SUA lowering effect of the milk proteins casein and lactalbumin. Interestingly, soy milk led to a 10% increase in the SUA level.

9. Regular consumption of coffee can help to lower SUA levels—in combination with proper diet and medication—and is therefore to be advocated.
  **Evidence: 2b (Grade B) | Level of agreement: 9.6.**

### Comment

This recommendation was classified as current and relevant and, apart from slight changes in the wording, was retained unchanged. The level of evidence has increased from 3 to 2b due to more recently available literature.

The mechanisms underlying the effect of coffee on SUA levels are not yet clearly defined. Several possible explanations are discussed. Caffeine (1,3,7-trimethyl-xanthine) in coffee inhibits xanthine oxidase, increases renal blood flow and improves urate excretion via the urinary tract. Both caffeinated and decaffeinated coffees contain chlorogenic acid, which can improve insulin resistance and thereby lower SUA levels. [48–51].

According to the literature, caffeine reduces the risk of gout [31, 32].

A correlation between coffee consumption and the risk of hyperuricemia is assessed differently. A meta-analysis from 2016 concluded that—with limited evidence due to a dearth of studies—coffee consumption may be associated with a lower risk of contracting gout, but at the same time points to a need for further well-designed studies. The results of a 2018 meta-analysis found no correlation between coffee consumption and SUA levels in men but an increased risk for women in this regard. Although regular coffee consumption does reduce the incidence of gout, coffee is not by itself an effective tool for reducing SUA levels but should only be considered supportive.

10. Cherries (especially the Montmorency variety) can lower SUA levels by promoting urinary excretion. However, it is still unclear at what dose the different products (juice, concentrate, extract) yield the most desirable effect. It is possible that sour cherries in combination with allopurinol have a complementary effect.
  **Evidence 2b (Grade B) Level of agreement: 9.0.**

### Comment

This recommendation from the 2014 report has been reformulated or replaces the recommendation to consume vitamin C, because the evidence for vitamin C use in this context must be classified as lacking relevance.

Among the plant foods with SUA-lowering potential, the most literature was found for cherries. Tart cherries contain particularly high amounts of anthocyanins and are considered to have many health benefits. Study data, especially on their potential of promoting urinary urate excretion by measurement are still contradictory at present and subject of an ongoing discussion [52–54]; however, the current albeit very sparse body of studies supports an association between the consumption of cherries and a consequently lower risk of gout attacks [55–58].

Further comprehensive studies are needed to evaluate the efficacy of cherry consumption in the treatment of patients with gout or hyperuricemia. Long-term effects and the exact mode of action for reducing gout flares are of special interest. Hence, the outcome of an ongoing study by Lamb et al. not yet published, remains to be seen. This study is the first randomized, double-blind, placebo-controlled trial to investigate the effectiveness of tart cherry juice in reducing the risk of recurrent gout flares [59]. The results of this important study could be decisive for further assessment.

## Discussion

There is already a robust body of evidence on the importance and the benefit of a healthy lifestyle including a balanced diet and physical exercise in people with RMDs, maintaining a healthy weight [60]. The impact of physical exercise and body weight control on lifestyle improvement in people with various RMDs is well known, also for gout patients [61]. There is also broad consensus that lifestyle improvements, although being an essential part of RMD management, can only complement medical treatment but do not replace it. Lifestyle recommendations, like medical treatment, should be tailored for the individual patient according to factors such as age, sex, health condition and comorbidities.

The aim of this study is to provide an evidence-based update of dietary and lifestyle recommendations for patients with gout and hyperuricemia based on selected literature available as of June 2021.

As with the initial recommendations provided in 2014, the authors decided to create joint recommendations for patients as well as physicians [11]. These combined recommendations are intended, on the one hand, to bring the current state of knowledge, including the level of evidence and relevant literature, to the attention of colleagues and, at the same time, to offer clear and comprehensible formulations and salient images optimized for patients. The choice of colors again corresponds to the generally used and internationally understandable traffic light system, i.e. red for agents to be avoided or reduced, green for those that are permitted and blue for general dietary and lifestyle recommendations. For optimal illustration, the structure is again divided into a written part on the front and a pictorial representation with icons (circular form) on the back, using colors identical to the written recommendations. The pictorial representation is also suitable for patients with language barriers. The order of the recommendations has been altered for style consideration: the three general recommendations with an emphasis on the importance of weight reduction and maintaining a normal weight range and the general favoring of a healthy diet were placed at the beginning. These are now followed by the four recommendations on foods and stimulants to be avoided or at least limited. Finally, there are the three recommendations of foods that should be promoted because they lower the SUA level.

The level of evidence has increased for five recommendations compared to 2014 due to more recent literature [11]. For the individual recommendations, evidence levels were tallied as once for 1b (recommendation 8, low-fat dairy products), seven times for 2(a/b), and twice for 3 (recommendation 5, seafood and 7, fructose). Paper selection was determined by members of the working group. The recommendations are based on authoritative publications from the available literature, whereby all prospective randomized studies on relevant topics as well as large epidemiological studies with accepted clinical relevance, including available meta-analyses and reviews were included. The hierarchical review process was carried out applying the Oxford GRADE system rules but without a methodological quality assessment for systematic reviews (e.g. assessment tool to assess systematic studies (AMSTAR)). This must be mentioned as a limitation of our work.

As with the initial recommendations from 2014, the focus was placed on practical relevance in order to meet the needs of patients and clinically active colleagues. The recommendations were deliberately not differentiated between gout and hyperuricemia, because both patient groups are meant to be addressed here, with the overriding goal of reducing SUA. Instead of the 9 recommendations offered in 2014, there are 10 recommendations in the updated version. Recommendations 3 (DASH diet) and 10 (Montmorency cherries) have been added. Vitamin C administration for gout patients is no longer included due to insufficient data [18]. Significantly, more recent aspects regarding weight management and the DASH diet have been taken into account here.

Significant results from studies in recent years show that controlled weight loss not only lowers both blood pressure and cardiovascular risk but can also be considered an important factor in lowering SUA levels via adjusted dietary habits, thus reducing the risk of developing gout [13–16]. Diet and lifestyle alone cannot cure gout, and this should not be suggested to any patient [62]. Urate Lowering Therapy (ULT) remains the mainstay of gout therapy, ideally complemented by correspondent changes of the individual lifestyle behaviour. Following the aforementioned recommendations by professional associations, diet and lifestyle recommendations should most certainly be included in the spirit of holistic patient management, alongside prescribed medication [63]. It is also important to emphasize dose dependency for all foodstuffs, beverages and stimulants mentioned, which is why an essential message is to avoid excess. One goal is to communicate to patients that foods and stimulants that increase SUA levels are not categorically banned forever, but can still be enjoyed, albeit in smaller quantities and less frequently. This is an essential strategy that may increase compliance more readily. In conclusion, the retention of eight of the original nine recommendations is evidence of the solid evidence base already present in the initial publication from 2014. The wealth of evidence on the DASH diet that has been added since the last publication in 2014 has been added in the form of an additional recommendation. Finally, the graphical representation was adapted to reflect inclusion of the new recommendations. The 2014 recommendations have already been translated into 10 languages (English, Arabic, Bulgarian, Chinese, Farsi, Croatian, Serbian, Romanian, Hungarian and Turkish) due to high demand in the field. A translation of the updated recommendations may be considered for the future.
